# Book Reviews

**Published:** 1832-12

**Authors:** 


### CRITICAL ANALYSES.

Quæ laudanda forent, et quæ culpanda, vicissim,
Illa prius, cretâ; mox hæc, carbone, *notamus.*—Persius.

*Elements of Materia Medica and Therapeutics; including the recent Discoveries and Analyses of Medicines.* By Anthony Todd Thomson, M.D. F.L.S. and G.S., Professor of Materia Medica and Therapeutics and of Medical Jurisprudence in the University of London, Member of the Royal College of Physicians, &c.—8vo. pp. 747. Longman and Co., London.

The task which Dr. Thomson has undertaken in the present work is one which is attended by no ordinary difficulties; for upon no other subject of medical literature is there so great a mixture of fact and fable as upon that which relates to the virtues of medicines, or the mode in which they act upon the animal economy. In order to produce upon these topics a work well adapted for the instruction of medical students, it is essentially necessary, on the one hand, that the author should jealously scrutinize the interested reports respecting the value of many medical agents, which are too frequently thrust upon the public and the profession; and, on the other, that he should be equally careful not to place too much reliance upon the statements of those who not unfrequently underrate the power of medicines, in consequence of their inappropriate and unskilful employment of them. Every step of the investigation which relates to the *modus operandi* of medicines tempts us to enter into hypothetical speculations, which would be quite out of place in an introductory treatise; for it ought to be the leading object of every elementary writer, and of every medical student, to adhere as closely as possible to well-ascertained facts, before any approach is made to what Burton quaintly terms the "upper stories" of science. In our opinion, Dr. Thomson has completely succeeded in avoiding the dangers which are incidental to the task he has imposed upon himself, and, after a careful perusal of the work, we feel justified in stating that it is one of the best and most valuable elementary treatises upon Materia Medica and Therapeutics which the student can devote his attention to, or the practitioner keep by him as a book of reference. To a certain extent, perhaps, we ought to qualify this expression of our favorable opinion, for as yet the *first* volume only of the work is published: but it is not very probable that he who has so ably gone through half his task will fail in his continuation of it. From what we know of Dr. Thomson, we do not fear that his industry will be diminished, and his experience must be daily accumulating: we have every reason, therefore, to anticipate that the second volume will at least keep pace with its predecessor.

After defining the terms *Materia Medica* and *Therapeutics*, and making some general remarks on *Medicinal Agents*, Dr. Thomson adverts to the general circumstances connected with the action of the latter on the living body. He enumerates five distinct modes in which medicines act upon the living body: first, they may act by a direct impression upon the surface to which they are applied, the effect being confined to the part; second, by an impression upon the nervous energy of the surface to which they are applied, and the effects be extended to the other parts of the system; third, they may be conveyed by absorption, undecomposed, into the system, and influence the habit through the medium of the circulation; fourth, they may be decomposed, and operate only by one or more of their constituents; fifth, they may operate by counter-irritation or revulsion. The direct and local action of medicines is illustrated in the influence which astringents, either taken into the stomach or injected into the rectum, exert in checking diarrhœa depending on a merely relaxed state of the intestinal canal; and in the effect of collyria applied to a bloodshot eye.

*Action of medicines upon the nervous energy.*

"That every medicine operates either directly or indirectly upon the nervous system can scarcely be denied. When a medicine is absorbed and taken into the circulation, we are unable to prove that any chemical change is effected in the circulating mass by it; and, even were this the case, still we must refer its ultimate effect to some action upon the nervous system, unless we suppose that the secretions are mere chemical changes in the fluids, altogether independent of the vital principle; an idea which is totally devoid of support. Chemical changes, also, in the fluids can be more readily explained, on the supposition that, a new action being excited in the organ or organs secreting the fluids, the result will be a change in the nature or the proportion of the components of these fluids, than that the medicine penetrates the vessels and acts chemically upon their contents. It is true that many medicines enter the blood-vessels, and, being conveyed to various organs, these are excited in the same direct manner as the surface to which the medicines were first applied: but, at the same time, phenomena occur which can only be referred to nervous sympathy.

"If we admit the principles that medicines can only affect the body through the medium of the nerves, that is, as far as regards their operation beyond the stomach or the part to which they are directly applied, there is no difficulty in understanding the manner in which the impression is propagated over the entire system, or to some organ perhaps the most distant from that which has received the immediate impression of the medicine. The physiological discoveries of Sir Charles Bell have so far unravelled the intricacies of the nervous system as to enable us to comprehend how impressions are communicated along certain sets of nerves, while other sets remain inactive; and, thence, to understand those associated changes which almost simultaneously occur in distant parts. From his investigations we learn that all the nerves of sensation communicate by originating in the same medullary tract, in the spinal marrow and brain; that a similar medium of communication unites the nerves of motion or volition, and a third also those of respiration. Now, if an impression be made on any portion of one of these sets of nerves, it is communicated to the whole set to which the affected nerve belongs, by the common medium which conjoins them. Thus, if a violent pain be felt in the great toe, as occurs frequently in gout, it may be allayed by a large dose of opium taken into the stomach. We do not suppose it necessary that the opium should be absorbed, and conveyed by the blood-vessels to the toe, in order to produce this effect; the impression of the opium is first received by the sentient extremities of the nerves of sensation of the stomach; the new disposition given to them is propagated to the connecting tract in the spine, and the sensorium commune, and thence to the part which is suffering. It is not my intention to enter into any metaphysical discussion respecting the real seat of the sensation of pain; it is sufficient to state the fact, that, by affecting the sentient nerves of the stomach, the opium allays a painful feeling depending upon a peculiar condition of the same set of nerves, in a distant part of the body. There is, then, a simultaneous action of some of the nerves in the most distant part of the body; indeed, it has never been doubted that those associated impressions, which we so often observe in disease, are to be referred to that simultaneous action. If this be a correct view of the subject, there is no reason why we should refuse to admit that the same power may regulate the operation of remedies. Let me state an example in illustration of this position. We are informed by a patient that he is suffering under palpitation of the heart; we find, on laying our hand upon the region of the heart, that this organ is labouring violently, and is in the most irritable state; and, yet, we can discover nothing which authorizes us to suppose that this disturbance depends, in any degree, upon organic disease or a change in the structure of the organ. We refer it, therefore, to the stomach, which we discover to be out of health, and we say that the heart associates, or, in other language, sympathizes with the morbid action of this viscus. No remedies are, in this case, administered with the view of directly influencing the heart; but those are prescribed which we know can correct the diseased condition of the stomach; and, as this organ becomes less irritable, the heart, also, becomes quieter, and recovers its healthy action." (P. 7.)

It may be asked, however, what proof we have that medicines act through the medium of the nervous energy, independent of absorption. The following facts are selected to support the opinion. Rhubarb, when taken into the stomach, is in some way introduced into the circulation, and easily detected in the urine; we have, therefore, no difficulty in ascertaining whether it has been absorbed. Now, if we apply a poultice made with a strong decoction of rhubarb to the abdomen of a child, purging is excited; but the presence of the medicine cannot be detected in the urine by the most delicate tests. If the skin of the hand be bathed with a strong decoction of tobacco, vomiting will be produced; but no absorption of the decoction takes place. Many experiments, particularly those of Dr. ROUSSEAU, of Philadelphia, prove that the power of absorbing is limited to a very small portion only of the surface of the body; the space, for instance, between the middle of the thigh and the hip, and that between the middle of the arm and the shoulder. Again, M. DUPUY, having divided the nerves of the eighth pair in a horse, two ounces of nux vomica, in the form of a bolus, were introduced into the stomach of the animal: no injurious consequences followed, although another horse, equal in size and strength to the former, to whom the same quantity of the poison was administered, died in a few hours, in violent tetanic convulsions.

"Upon the whole, there is abundant evidence that many medicines produce their effects by acting directly on the nervous energy, altogether independent of absorption. It is not essential that we should be able to demonstrate in what manner this communication with distant parts of the body is effected by the nerves; it is enough to know the fact, and we cannot proceed farther. The attempt to explain the phenomenon is as vain as that respecting the vital principle; we find ourselves out of our depth, and the struggle only convinces us that we are in an element which does not belong to us.

"In whatever way this nervous communication is accomplished, there are three surfaces upon which the impressions can be advantageously made, and whence they are propagated: the stomach and alimentary canal, the skin, and the organ of smelling." (P. 10.)

The most curious circumstance connected with the operation of medicines taken into the stomach, is the selection, as it were, by the medicine, of the part of the system on which it shall act: thus, a diuretic taken into the stomach exerts its influence on the kidneys; an expectorant, upon the mucous membrane lining the trachea and the bronchial tubes; and a diaphoretic, upon the skin. Dr. Thomson states, as a general proposition, that larger doses of medicine can be thrown into the rectum with impunity than into the stomach. We believe, however, there are some exceptions to this statement, and we may mention opium as one. Baron DUPUYTREN and others have recorded several cases from which it appears that very small quantities of laudanum injected into the rectum produced much more powerful effects than if the same dose had been taken into the stomach. Upon turning to page 532 of the work, where Dr. T. treats particularly of opium and its mode of action, we find the following passage, in reference probably to the cases to which we allude.

"It has been asserted that opium exhibited per anum has a much greater influence upon the habit than when it is taken into the stomach; and this is said to be owing to the gastric juices altering the nature of the medicine and weakening its narcotic power; whilst in the rectum no such change occurs, and if the gut be not loaded with fæces, the opium is rapidly carried into the habit. But this reasoning is more specious than true: the gastric action tends merely to disengage more quickly the morphia, which is indigestible, whilst no such evolution takes place in the rectum. Thence, notwithstanding the results of some recent cases, much larger doses of opium can be administered by the rectum than by the mouth." (P. 532.)

Still facts are stubborn; and we must suggest the necessity of caution in administering opium by the rectum.

After giving a brief, yet satisfactory sketch of the action of medicines upon the nervous system, Dr. T. passes to the action of medicines which are absorbed in their entire state. A medicine may be conveyed undecomposed into the circulation, and influence the general system, either by absorption from the intestinal tube, by absorption through the skin, or by absorption through the lungs. Sufficient proofs are noticed to shew that medicines are absorbed from the intestinal canal. The absorption of medicines by the *skin*, it is confessed, is less easily demonstrated. Dr. T. refers to the observations and experiments of many celebrated physiologists, who have directed their attention to this point; and he finally states it as his belief, that there is little doubt that absorption occurs to a certain extent, but he considers it questionable whether this is a function of the whole skin or of a part.

*Absorption of medicines through the Lungs.*

"Some experiments of Dr. Roussean are sufficient to prove that turpentines, musk, garlic, camphor, and some other volatile substances, are very rapidly absorbed by the lungs, and taken into the course of the circulation. There is no reason for supposing that any decomposition of the medicines occurs in these instances. Whether gases are absorbed, is uncertain; indeed, it is difficult to conceive how they could be condensed, and carried undecomposed through the course of the circulation. Although the air is the vehicle by which volatile substances are conveyed into the lungs, and although it undergo a chemical change in the bronchial cells, yet this does not affect the volatile matters held in solution by it when it is inspired: these are taken up by the absorbents opening on the mucous membrane of the bronchial tubes; whence they are carried into the circulation, and again excreted by the kidneys and the skin. In their passage, however, they powerfully stimulate the nervous system, and occasionally produce as decided an influence on the body as if they had been taken into the stomach. An excellent illustration of this fact is observed in those persons who are employed to pump ardent spirits from large casks into small vessels for the convenience of the retail dealer. If this operation be performed in a cellar, or in any confined place, the assistants become intoxicated, without taking the spirits into the stomach. It might be supposed that, as the whole body, in these cases, is immersed in an atmosphere highly impregnated with the volatile matters, the skin and the lungs may be regarded as the inlets; but there is no direct proof that, even when the cuticle is removed, ardent spirits can be taken up by the absorbents of the skin, nor even by the lungs; whereas the proof is direct, that they affect the system and produce intoxication by acting on the olfactory nerves.

"The difference between the mucous membrane of the bronchial tubes and the skin or cutis, as I have already informed you, consists in the medium interposed between them and the air: in the skin this medium is the cuticle; in the lungs it is a fine epithelium, which certainly is more likely to permit imbibition than the cuticle. It is possible that in this manner, and not, strictly speaking, by some true vascular or lymphatic absorption, some volatile matters admitted into the lungs are conveyed into the circulation." (P. 17.)

The subjects next considered are the action of medicines decomposed in the stomach, or after entering the system; the revulsive or counter-irritant action of medicines; the chemical action of medicines; and the general effects of medicines on the vital solids and fluids, and on the functions.

"*Effects on the living Solid.* It is not easy to define what the living solid implies, nor is it essential for our purpose. It is sufficient to know that the term is employed to express an indefinite idea of what we suppose to be the ultimate fibril of the organic tissues, whether cellular, muscular, or nervous; and it is probable that the change in the condition of any organ, produced by a medicinal agent, is owing to some change in condition or arrangement which the medicine impresses on this fibril. Thus the action of the heart is augmented after the dose of alcohol has been taken into the stomach: we can form no other idea of the cause of this, unless we suppose that either the nervous fibrils in the stomach, from the condition of the alcohol, undergo some change in their condition, which is followed by a corresponding change in the contractile fibres of the heart; or that the alcohol carried into the circulation is applied to the tissue of the heart, and effects the same change by the immediate application of its particles to the ultimate fibrils of which that tissue is composed. But neither the existence of the fibrils, nor the nature of the change which they suffer, is capable of demonstration: we found our belief of both solely upon the effects which we observe invariably to follow the internal administration of a dose of alcohol. These are a sensation of warmth in the gastric and thoracic regions, a more forcible action of the heart, and an increased momentum of the blood, inferred from the state of the pulse, compared with that which existed before the alcohol was taken. All medicines producing effects similar to those referred to the alcohol we regard as stimulants; whilst those, the administration of which is followed by diminished action, a preternatural decrease of the general momentum of the blood, and a lowering of the animal temperature, are regarded as sedatives. The general primary effect of every medicinal agent upon the living solid may be, therefore, said to be either stimulant or sedative. This, however, is not the prevailing view of the subject: the primary influence of every medicine on the living solid is regarded as stimulant; whilst the sedative effect is supposed to be altogether negative, the result of the prior stimulant impression: or, in other words, that any sedative effect which follows the administration of a medicine is that state of collapse which always succeeds the stimulant action of substances that operate directly on the nervous system. This reasoning is undoubtedly in perfect accordance with the law of the system, that all impressions made upon nerves have a direct tendency to exhaust their energy, and an indirect tendency to lower the power of the system in general: but, nevertheless, the same train of reasoning which admits of the conclusion, that the effects which follow the administration of certain medicines are the result of a stimulant impression on the living solid, authorizes, on the other hand, the assertion, that the diminished momentum, and other phenomena indicative of an abstraction of power, which immediately succeeds the administration of other medicines, are as truly the result of a positive change of constitution in the living solid which may be termed sedative. Other arguments might be advanced in support of this opinion; but, as it must again be discussed in treating specially of sedatives, the further consideration of it is unnecessary at present." (P. 27.)

With respect to the effect of medicines on the blood, Dr. T. remarks, that the blood may be mingled with medicines which have been taken into the stomach, and have passed into the circulation, either in whole or in part; but during life he sees no reason for believing that any material alteration, even of the physical properties of the blood, occurs from this cause. "Some late experiments of Dr. Stevens on the effects of saline substances upon the blood, seem to be at variance with this opinion; but these are too little understood to be brought forward as opposing the opinion which has been advanced."

*Circumstances modifying the general action of medicines.* Many and different circumstances tend to modify the operation of medicines. Some are connected with the original conformation of the body; others with the age and the sex of the individual; some with the situation on the face of the globe in which he is placed, as influencing his system by diet and habit; others, again, with the state of society, its customs, superstitions, and even practical relations; and, lastly, some with the condition of the mind, displayed in temper and intellectual attainments. Upon each of these points Dr. T. offers many interesting remarks. In those whose nervous systems are exquisitely susceptible to impressions, not only external agents and powerful mental affections interfere with and modify the operation of medicines, but, in such a condition of the nerves, even volition exerts an extraordinary sway, working either for or against their efficacy, and sometimes completely counteracting their usual effects. The following anecdote is mentioned by Dr. T., to illustrate the control of the mind over the operation of medicines, in such states of the constitution. A lady was labouring under an affection of the bowels, attended with severe pain and obstinate costiveness. She was bled; the warm bath and fomentations were frequently resorted to, and purgatives and various anodynes freely given; but without the least effect upon the bowels, and without either sleep or any diminution of pain being procured. At length the physician in attendance was informed that she had expressed her conviction that her usual medical attendant in the country alone understood her constitution, and was the only person who could relieve her: this gentleman was accordingly sent for; and although no change, either of measures or of medicine, was resorted to, yet the bowels were quickly moved, sleep and a cessation of pain followed, and in a few days the patient was convalescent. Many cases of a similar kind have fallen under our own observation.

Dr. T. mentions many facts tending to prove how powerfully idiosyncrasy may modify or completely alter the usual action of medicines; and to this very important subject the student should pay especial attention; and the physician, in prescribing medicine for the first time to any patient with whose habit he is not well acquainted, should endeavour to ascertain what effect it may have produced when previously administered. The effects of age and sex in modifying the action of medicines should also be well known to the practitioner: in different periods of life, the same medicines produce very distinct effects.

"In the female, whatever may be the cause of that periodical determination of blood to the uterus which is termed menstruation, or whatever may be its ultimate intention, its presence or its absence produces a very considerable difference in the operation of various medicines. Thus, preparations of iron, which generally operate beneficially in rousing the energy and promoting the tone of the system in pale leucophlegmatic females, when the menstrual discharge is either obstructed or deficient, would prove deleterious in strong young women, in whom this excretion is regular and in sufficient quantity. In pregnancy, many diseases may supervene; but the remedies which relieve similar diseases in females who are not pregnant, cannot always be employed with safety. In the unimpregnated female, the irritable state of the stomach, which is accompanied with heartburn and vomiting, may be judiciously managed with sedatives; such, for instance, as the hydrocyanate of potassa; but, in the pregnant state, as this salt, when given to the mother, has been detected in the fœtus, much injury may accrue to the unborn infant from its administration, either in full or frequently repeated doses. Opium, also, when given in large doses, if frequently repeated, for the relief of cramps or inquietude in the mother, is ultimately injurious to the fœtus. To the mother, also, these medicines may prove hurtful, from their action being modified by the disturbed state of the functions of the brain, which so often occurs during the period of pregnancy. For the same reason, some stimulants which may prove highly beneficial in palsy, in the ordinary state of the body, often increase the complaint, and even tend to produce a fatal termination of that affection, in the pregnant female. The operation of many other medicines are equally modified by this state; and, indeed, no violent medicines can be safely prescribed for a pregnant female; nor should the hot bath ever be used in pregnancy." (P. 52.)

*Effects of Custom in modifying the action of medicines.* The influence of habit, or custom, over both the mental and corporeal functions in man, is remarkable, and its control over the action of medicines is not less wonderful. In the section devoted to this subject, Dr. Thomson brings forward many facts to shew the extent to which the effects of medicinal agents are thus modified or altered. The general inferences to be drawn from these facts are the necessity of augmenting the doses of medicines, when it is requisite to continue them for some time; and also of occasionally suspending, for a short period, the use of a medicine, so that, when it is again employed, the impression may be renewed with sufficient energy.

*The influence of Climate* in modifying the action of medicines, operates in two ways: 1st, by the change which climate causes in the animal frame; 2d, by the change which it occasions in medicinal agents of a vegetable origin. Dr. T. first points out the striking influence of climate in producing various alterations in the aspect and habits of the human species, and on the tribes of the lower animals: it will hence be easy to form an idea of the power which climate is likely to exert in modifying the operation of medicines.

"The late Dr. Harrison found that narcotics act with greater force, even in smaller doses, in Naples than in England. He instances the extract of henbane, which, in doses of three grains, thrice a day, at Naples, produced a temporary amaurosis or nervous blindness, which disappeared and recurred on the alternate suspension and administration of the medicine. This effect of the extract was observed in two patients, who had often taken similar doses of the same remedy in England, without any unpleasant result; an effect which Dr. Harrison correctly refers to the increased nervous susceptibility of impression of the patients, in the warmer climate. It might be supposed that the Italian extract is more powerful than the English; but that argument would not apply in this instance, as the medicine which was administered in Italy was procured from London; consequently, any increase of its power could not be attributed to the influence of climate on the medicine itself. Dr. Harrison found, also, that nitrate of silver and nitrate of mercury are more active in Italy than in England; and that, in general, the doses of medicines ordered in this country are too large for the climate of Italy. It does not, however, always follow that the doses of medicines require to be reduced in warm climates: on the contrary, in India, a scruple of calomel and a grain of opium are frequently administered, and repeated at short intervals, after depletion in dysentery; but few physicians would venture to prescribe this active remedy, in such large doses, in this climate.

"But even the state of the weather and the season of the year will, sometimes, alter the action of a medicine, in the same country. Thus, Mr. Annesley, in his work on the diseases of India, informs us that, in the subsidiary fever of Nagpore, the cinchona bark, although the grand remedy in the cold season, yet generally fails in the rainy season; at which time calomel and antimony prove beneficial.

"The neglect of observing the effects of the influence of climate in modifying the action of medicines has led to many of the discordant accounts of remedies by different writers, and the rejection of many valuable medicines. Many medicines, also, have been unjustly depreciated from having been administered at improper seasons of the year, or from no allowance having been made for the power of local circumstances over their action in the animal economy. An example of this description is found in the Rhododendron Chrysanthum, which has been very successfully employed for the cure of rheumatism in Russia, but has greatly disappointed the hopes of practitioners in this country. This failure has been attributed to the plant suffering from drying and export; but the cause is more likely to be found in the effect of climate upon the constitutions of those to whom it is administered. We may, also, refer to the same cause the greater efficacy of guiaicum in Hispaniola and other warm climates, in curing syphilis, than in Europe. In treating of the different articles of the Materia Medica, I shall have frequent opportunities of illustrating the truth of this remark. In prescribing for those who have lately arrived in a country of an opposite climate to that from which they have come, and who have not had time to be naturalized to it, the knowledge of the above-mentioned facts will be found useful. Thus, in the case of a person who has arrived in a hot from a cold climate, the susceptibility of impression being greatly augmented, the habit acquires a febrile tendency, and will not admit of the same doses of stimulant medicines that may be given with advantage to natives of the place, and to those accustomed, from long residence, to the climate. Such are the effects of climate upon the animal frame, and its consequent influence on the operation of medicines." (P. 69.)

Climate also produces changes upon the vegetable tribes as striking as those upon the animal, and consequently medicines of a vegetable origin have their active powers more or less changed, if removed from the spots where nature had planted them, to be cultivated in foreign soils. This is, indeed, an almost insurmountable obstacle to the naturalization here of medicinal plants of latitudes greatly different from that of England: in general, the virtues of the plants are diminished, if not totally destroyed, by the transportation.

"The effect of climate upon the medicinal properties of plants is strikingly illustrated in the history of the meadow saffron, colchicum autumnale. In England, and many other countries, the colchicum always contains an acrid, alkaline, bitter principle, Veratria, of great importance as a remedy, and a virulent poison when overdosed. At some seasons of the year, the bulb of the colchicum, in this country, is more active than at others; but, during the whole year, it contains a sufficient portion of its medicinal principle to render it a very hurtful substance, if eaten as food: yet in other parts of the globe it may he eaten with impunity at some seasons. Kraterhvill, a German author, in his work 'de Colchico,' relates instances in which entire bulbs were eaten without any bad effect being produced on the habit. Krapf, another German writer, says that he has eaten the bulb with impunity in autumn, and that it is then eaten in Carniola and Istria: yet autumn is the period of the year when the bulb is most active in this country. The celebrated Haller, also, avers that it is both tasteless and inert in autumn. Now to what are we to ascribe those peculiarities in the colchicum of the countries in which these writers lived? Their veracity is undoubted; yet their relations are at direct variance with our experience in this country, and, therefore, we can only ascribe the difference to climate and to local circumstances. For the same reason, senna transported from Upper Egypt, and grown in the south of France, varies both in the external characters of the leaf and in its purgative properties: the leaves are more obtuse, less bitter and less nauseous when chewed, and much less purgative than the Egyptian senna. The tree named Myrospermum Frutescens, when it grows in New Grenada, yields balsam of Tulou; but when it grows in Peru it yields a very different balsamic substance; which, from the place of its production, has been named Balsam of Peru. I may also mention that mint, and many other plants which yield an essential oil, afford it of a much less penetrating odour in the south of Europe than in England: and it is a curious fact, that almost all strong smelling plants lose their odours in a sandy soil.

"From these facts, it is very obvious that the nature of the climate in which medicinal plants are cultivated should be known. Indeed so very important is it that medicines should always be as nearly as possible the same, that a medicine coming from any other part of the world than that from which it was originally obtained ought not to be trusted in the cure of diseases, until a set of comparative experiments have determined its affinity in every respect to the original drug." (P. 71.)

*Cultivation* has a close resemblance to climate in its effects upon the medicinal properties of plants. "Few plants which are medicinal admit of cultivation, although edible vegetables are greatly improved by it." Other circumstances also contribute to vary or alter their powers: thus, all medicinal plants do not acquire their active qualities until they have attained what may be termed adult age; even poisonous plants may be eaten with impunity when they are young.

"Some plants, again, have their active principles suddenly developed at a fixed period of their existence: the lettuce, for example, when young, possesses scarcely any of the narcotic principle which constitutes the Lactucarium of the Pharmacopœias, although it is abundantly secreted at the flowering season. In the poppy, the narcotic principle is scarcely apparent until the petals fall and the germen enlarges. The soil also, its dryness or its moisture, the degree of exposure of the plant to heat, light, and air, all contribute to modify its medicinal qualities; and thence it happens that plants collected in one year may display great activity, whilst in the next they may appear almost inert. A plant which grows naturally in a dry or an absorbent soil is generally less active when it is found growing in a humid or a marshy situation: another which is the ordinary inhabitant of an exposed spot, and requires the invigorating influence of the stimulus of much light, heat, and air, languishes and loses its medicinal virtues when it rises accidentally in the forest; whilst others, again, only acquire them in the shade. It is, indeed, these circumstances, in a great measure, which distinguish climates, and which augment the remedial properties of vegetable bodies that owe their activity to volatile oil, resin, and the balsams. The knowledge of these facts is of great importance to the collectors of medicinal plants; who should be able to determine the period in the life of a plant, the nature of the soil, the degree of exposure, and the season of the year most favorable to the development of active properties." (P. 73.)

*Influence of Mental Affections in modifying the action of medicines.* The powerful influence of mind over the functions of the body, is well known to every observing physician. The facetious author of "Tristram Shandy" strongly expresses this fact, when he compares the body and soul to a coat and its lining: "if you rumple the one, you rumple the other." This influence is exerted according to the nature of the passions, which are arranged in two classes, the *depressing* and the *exciting*. All experience in the practice of medicine tends to convince us how necessary it is to be aware of the influence of these passions on the system of the patient; not only at the moment when the physician is about to prescribe for him, but in observing the effects of the medicines prescribed. Dr. Thomson points out the modifying influence over the action of medicines of various mental emotions, as vexation, sorrow, fear, terror, joy, confidence, &c.

*Influence which the Period of a Disease exerts over the action of medicines.* It is easy to conceive that many circumstances connected with the progress of disease (the changes, for example, in the nervous irritability, in the force of the circulation of the blood, and in the temperature of the body,) must tend to render the administration of a medicine which acts beneficially at one time less beneficial at another.

"If a drastic purgative be given soon after an ague has been checked by tonics, it is probable that the disease will return; a remark which was noticed by Sydenham and De Haen, both high authorities in all practical matters. Thus, also, at the commencement of dysentery, whilst inflammation of the mucous membrane exists in the large intestines, it would be extremely hazardous to administer stimulants, tonics, and astringents; but, when the inflammatory symptoms have abated, when the debility which is the result of that state alone threatens the life of the patient, then tonics and astringents are not only admissible, but they are absolutely required. In some diseased conditions of the habit, a common purgative may prove even fatal, if administered at an improper time, independent of that state of great corporeal debility, which would prevent any sensible practitioner from prescribing a medicine the operation of which would only add to the already too greatly exhausted state of the body. Thus, when accumulations take place in the bowels, from mechanical causes, such as hernia, intussusception, or permanent contractions, fatal results have followed the administration of a violent cathartic: and equally dangerous might prove the administration of cathartics whilst the intestines are in a highly irritable state, either from inflammation, ulceration, or an inordinate determination of blood to them from any cause.

"The influence of the period of disease, in controlling the operation of medicines, might be illustrated by many other examples, were it not premature to bring them forward at the present moment. I will merely, therefore, mention one or two more in support of my position. In dropsy, if foxglove be given early, whilst the pulse is hard, quick, and incompressible, it produces no beneficial effects, the functions of the capillaries are not increased, nor is the secretion of urine augmented; but, if the excitement be first reduced, whether by bleeding or any other means, the remedy then fulfils the indication of its administration; it lowers the pulse, unloads the capillaries, increases greatly the secretion of the kidneys, and relieves the serous sac of the superabundant fluid which has been deposited in it. If the action of the same medicine, foxglove, whether administered in dropsy or in phthisis, be not closely watched, the medicine appears to accumulate in the system, and dangerous results supervene; but, in other states of the habit, (in insanity, for instance, accompanied with great watchfulness,) the remedy may be carried to an extent far beyond that which is safe under any other circumstances. I have given an insane patient sixty minims of the tincture of foxglove every sixth hour, for several successive days, with no other effect than that of procuring quiet and sleep. It is necessary, however, to state that this dose was not given until the medicine had been administered for upwards of a week, in gradually augmented doses. In general it is proper to begin with ten or fifteen minims; after which the dose may be increased five minims each alternate dose until sleep be procured. When this has been effected, the dose must then be diminished in the same ratio in which it was augmented.

"Many salutary processes, also, occur in the progress of disease, which should not be checked by an improper administration of medicines. Thus, violent shiverings and tremors of the body, unaccompanied with coldness and not followed by preternatural heat, occasionally relieve acute gouty pains; but when these are interfered with, metastasis or a translation of diseased action occurs, and the inflammation, instead of remaining in the toe or the instep, attacks the head or the stomach. In the administration of local remedies, also, much caution is requisite; and every day's experience teaches us why many local diseases cannot be removed, or even checked, by local remedies, without the hazard of converting topical into general disease, or causing what may be termed a constitutional effort in some other part more essential to life than that which the attempt was made to relieve. In the administration of some internal medicines, also, the result may be a check to the salutary action of some local disease on the system, and thus, for a temporary and delusive suspension of present suffering, the most serious evil may follow. Thus, the shivering which often attends the passing of a gall-stone is supposed to operate like exercise, and to aid in the propulsion of the extraneous body. Even convulsions sometimes may be regarded as a salutary process. Dr. Parry mentions the case of a young lady who was long afflicted with headach, vertigo, and vomiting, which at length ended in total blindness, so as to induce a belief that she laboured under hydrocephalus internus; all the symptoms were, in a few hours, removed by a violent fit of convulsions. I have mentioned these instances to shew that the knowledge of the powers of a remedy does not constitute all which the physician ought to possess previous to prescribing it, even supposing he has taken into account all the circumstances which have been described as likely to modify its effects; he must, also, be convinced that no danger will result from its salutary influence in one part producing a translation of diseased action to another previously in a healthy state." (P. 88.)

We have now concluded our analysis of the first part of the work; the second and third parts are equally important, but our limits prevent us from entering upon the subjects which they embrace. The second part is divided into four sections, and treats of the *Classification of Medical Agents*; the third parts comprises a *History of Materia Medica and Therapeutics*, and in this division of the work we find abundant evidence of the judgment evinced by the author in the impartial statements he gives of the virtues of medical agents, and their effects upon the constitution. Dr. Thomson's observations and cautions upon the subject of blood-letting, and the administration and effects of opium, are especially worthy the study of the medical pupil. From the remarks Dr. T. offers upon "the action of narcotics on the nervous system," we find that he places implicit reliance upon the results of the experiments made by Messrs. MORGAN and ADDISON.* In the review

* Essay on the Operation of Poisonous Agents on the Living Body.

we gave of the work of these gentlemen in our Journal for February 1830, p. 133, we stated it as our opinion that their experiments were inconclusive, and the results fallacious. We think so still; and if Dr. T. will refer to the number of our Journal we allude to, we believe he would become a convert to our opinions in reference to these experiments, and that he would agree with us in regarding them as less "demonstrative" than he states them to be at page 500 of his work.

---

*On the Influence of Physical Agents on Life;* by W. F. Edwards, M.D., F.R.S., Member of the Royal Academy of Sciences, and Royal Academy of Medicine of Paris, &c. *Translated from the French,* by Dr. Hodgkin and Dr. Fisher. *To which are added, in the Appendix, some Observations on Electricity,* by Dr. Edwards, M. Pouillet, and Luke Howard, F.R.S.; *on Absorption, and the Uses of the Spleen,* by Dr. Hodgkin; *on the Microscopic Characters of the Animal Tissues and Fluids,* by J. J. Lister, F.R.S., and Dr. Hodgkin; *and some Notes to the Work of* Dr. Edwards.— 8vo. pp. 488. Highley, London.

We have here, within the compass of one volume, and that one not over voluminous, as much matter condensed as would suffice for many of the modern (so called) "*libraries.*" Drs. Hodgkin and Fisher have indeed, in their translation of Edwards' memoir "on the Influence of Physical Agents on Life," with the appendix, and notes which they have added, brought together a vast number of curious and important facts, connected with a most curious and important subject.

The influence of physical agents on life, now that attention has been turned to them, will be acknowledged, on all hands, to be most general and important. If the influence of medicines be great, (medicines which are only taken occasionally, and by spoonfuls,) the influence of food, which is taken oftener, and in larger quantities, must, as dieticians have already observed, be far greater; and if the influence of food, which is taken only periodically, be so great, the influence of the physical agents, such as heat, light, electricity, atmospheric moisture, and the air we breathe, must be greater still: and so, in truth, the influence of these physical agents is, and their influence has only been hitherto unobserved, because it is so constant that we cannot withdraw ourselves from it, and live; only so long been so little thought of, because, from their nearness, their magnitude was unperceived.

But, to estimate the influence of these physical agents truly was no easy task; although, from the beginning of the investigation, it was evident that, when known, their investigation might greatly conduce to the preservation of health, and greatly facilitate its recovery when lost.

For some years Dr. Edwards has devoted himself to the inquiry, and the progressive fruits of his researches have been from time to time communicated to the Academy of Sciences at Paris, and subsequently to the philosophic world at large. Many of these original memoirs have been translated into our own journals at length, and most have had their more important features extracted; circumstances which prove the estimation in which this physiologist's observations are held, both here and elsewhere; and to the same point the members of the Academy have borne willing witness; for, as Dr. Hodgkin observes, they "obtained for their author, although a foreigner, the honourable distinction of the physiological prize."

Such having been the course of publication of these papers, the truths which they reveal have, many of them, been long received as canons in physiology, and incorporated into the more modern system in most countries; and therefore to them we shall not now particularly refer, more than to express our satisfaction in finding them collectively published, and especially presented, as they now are, to the English reader in an English dress, with the notes and annotations of the present editors.

It would be utterly impossible, within the legitimate compass of a review, to follow this work stage by stage: those of our readers who are particularly interested in these inquiries we refer to the volume, promising them much pleasure in its perusal. We shall confine our extracts chiefly to the Influence of Heat and Cold, and of Light on Animals, and to the chapter on Respiration.

"*On the Heat of Young Animals.* It is a general opinion, inferred from the circulation being more rapid and the nutritive functions more active in young animals, that their temperature is likewise more elevated than that of adults. But this opinion not being founded upon direct observation, I turned my attention to it at the commencement of my researches on animal heat. By means of a thermometer placed under the axilla, and the bulb applied so as to be on all sides in contact with the animal, I ascertained the temperature of some new-born puppies whilst in the act of sucking, and found it to be nearly equal to that of the mother, about a degree or two lower; but as this difference is not constant, and is observable among adults also, it may be altogether disregarded. We are therefore warranted in concluding that the temperature of the new-born animal, when placed near its mother, is not superior to that of adults.

"But if, at a temperature between 10° and 20° cent., or 50° and 68° Fahr., a new-born puppy be removed, and kept an hour or two from its mother, its temperature falls considerably, and continues falling, until, in the course of three or four hours, it stops a very few degrees above that of the surrounding air.

"This effect cannot be occasioned by the want of food for so short a time; and, even though it were, the difference in this respect between young and adult animals would be no less remarkable. But the temperature begins to fall as soon as the separation takes place, and the diminution is not in the least retarded by furnishing the young animal with milk from time to time. The same phenomenon takes place with kittens and rabbits.

"It might be supposed that this difference is accountable from the difference in the natural coverings; as rabbits, for example, are born almost naked, and certainly cool more rapidly than puppies and kittens; but, on the other hand, these, although well covered with hair, will cool down to the same degree, though more slowly; so that this circumstance can have but a secondary effect. Besides, the substitution of an artificial covering is found only to retard, not to prevent the lowering of the temperature to the same degree. We must therefore admit that, in the young animal, less heat is produced in a given time than in the adult.

"If we examine the change which the temperature undergoes in the process of life, we shall find at first but little alteration; after a while the diminution will take place more slowly; then the limit to its descent will be gradually higher and higher in the scale, till, at the end of about a fortnight, it will maintain itself at a degree nearly equal to that of the adult animal.

"The remarkable change which takes place in the young of the mammalia, with respect to their temperature, makes them pass from the state of cold-blooded to that of warm-blooded animals.

"The phenomena above mentioned are not, however, common to the young of all the mammalia. The heat of young guinea-pigs, born when the temperature of the air is between 10° and 20° cent., or 50° and 68° Fahr., in the above experiments, will be found to be nearly as great as that of adults, and if they be separated under the same circumstances, it is not diminished. The same is true of many other animals of this class. The young of mammalia appear to be distinguished into two groups in relation to animal heat. Some are born, as it were, cold-blooded; others warm-blooded. Corresponding with this difference, is a distinction deducible from the state of the eyes. Some are born with the eyes closed, others with the eyes open. The temperature of the former, according to the foregoing experiments, rises successively, and at the end of a fortnight, (which is the period when the eyes open), it is nearly equal to that of adults. Thus, the state of the eyes, through having no immediate connexion with the production of heat, may yet coincide with an internal structure influencing that function, and certainly furnishes signs which serve to indicate a remarkable change in this respect, since, at the period of the opening of their eyes, all young mammalia have nearly the same temperature as adults." (P. 58.)

That fish cannot live in water wholly deprived of air, has long been known; but Dr. Edwards has shewn, by a series of very conclusive experiments, that they can sustain the absence of air for a much longer period in cold than in warm water, and that, the higher the temperature of the medium becomes, the more speedily they are destroyed. An admirable provision this in their constitutions; for hence, when ponds and lakes are frozen over, and the access of air to the water, or of fish to the air, prevented, the necessity for it, and its consumption, is gradually diminished.

"Comparative experiments were made on individuals of the same species, and with as close a resemblance as possible, at temperatures varying from 0° cent. or 32° F. to 40° cent., or 104° Fahr. The result was, that at the higher limit death was speedy as with the batrachians, and the duration of life progressively augmented in proportion as the temperature was diminished to the lower limit. It is here seen that the effect of temperature (excluding all other influences) is altogether analogous to what has been observed in the batrachians; that the limits of the shortest and longest duration of life in the batrachians and fishes, placed in water deprived of air, are alike in both; and that in the same range of temperature, from 0° cent. or 32° F. to 40° cent., or 104° Fahr., the duration of their life goes on augmenting or diminishing, according as the temperature falls or rises between these extremes.

"In regard to the differences which fishes of the same species present at the same degree of temperature between these limits, size has a marked influence, the smallest as well as the youngest are those which are the least capable of bearing an elevatiou of temperature. However different may be the duration of the life of small fishes at low temperatures, at 40° cent., or 104 Fahr., it is almost uniform in all: they scarcely ever live more than two minutes; but the larger fishes are able to survive several minutes longer.

"Sect 2. *Influence of the Temperature of Aerated Water, in limited Quantities, in close Vessels.*

"On varying, in a series of experiments, the temperature and quantities of aerated water, it appears,

"1st, That the duration of life goes on increasing with an increase of the quantity of aerated water, the temperature remaining the same.

"2. That the same result takes place when, the quantity of water remaining the same, we lower the temperature.

"3. That the duration of life remains the same, when, within certain limits, we increase or diminish, at the same time, both the temperature and the quantity of aerated water."

"Sect. 3. *Influence of Temperature, and limited Quantities of Aerated Water, in contact with the Atmosphere.*

"Sylvestre has ascertaiued that a limited quantity of aerated water, in which a fish is placed, absorbs the air in contact with its surface. It evidently follows from this fact, that the life of the animal, in a limited quantity of water, will, cæteris paribus, be the longer, the more fully the absorption of air by the water compensates for that which the animal consumes in the water. Add to this, that the fish, when free, is able to derive directly from the atmosphere fresh supplies of air, according to its wants.

"Let us now see the influence of temperature under these circumstances. Take for example a bleak (Cyprinus alburnus). If we put it into a vessel with a large mouth, containing five ounces of aerated water at 20° cent., or 68° F., in summer, it dies within a few hours: but when the temperature is lowered to 10° or 12° cent., or 50° or 53° F., and is kept at that degree, the animal lives until its secretions are so abundant as to corrupt the water. If, to remedy this inconvenience, we merely renew the water every twenty-four hours, the animal lives in it almost indefinitely.

"This is exactly what we have seen to take place with the batrachians. Between 0° cent., or 32 Fahr., and 10° or 12° cent., or 50° or 53° Fahr., they live an indefinite time in aerated water, provided it be renewed sufficiently often; but they die for the most part as soon as the temperature rises above this limit.

"Let us now examine the general result of these facts, notwithstanding the different conditions in which the animals are placed. The more the temperature is raised beyond certain limits, the greater is the degree of the influence of the air required for the support of life. This influence, without reference to other causes, will be great in proportion to the quantity of this fluid. Here, however, there are limits depending on the organization of the animal." (P. 56.)

These latter observations shew that an increased temperature, above a certain limit, is inimical to fish and the batrachian reptiles; but that both animals and plants will, when gradually inured to such changes, bear a very high degree of temperature, has been often noticed by naturalists; and Dr. Hodgkin has collected several of these cases in an interesting note, from which we shall make some extracts.

"I have been furnished with the following authorities for fishes being able to live in water at very high temperature, by my excellent and accomplished friend, A. R. Dusgate, of Paris, from which it would appear that, in a state of nature, fish not only live, but thrive, in a temperature beyond the limit which they were able to endure in Dr. Edwards' experiments: may not this difference be in part referred to the influence of habit? Saussure, speaking of the hot springs of Aise, in Savoy, says, 'I have frequently examined the temperature of these waters at different seasons, and have always found it very nearly alike, viz. from 35 in that of Souffre, and from $36\frac{1}{2}$ to $36\frac{7}{10}$ in that of St. Paul. Notwithstanding the heat of these waters, living animals are found in the basins which receive them. I saw in them eels, rotifera and infusoria, in 1790. I discovered in them two new species of tremelles possessing spontaneous motion, of which a description may be seen in the 'Journal de Physique, for 1790, p. 401.'—See Saussure, Voyage dans les Alps, vol. vii. pp. 11 and 1168. Neufchatel edition, in 8vo.

"Sonnerat states, that in the island of Lugon, one of the Manillas, there is a hot spring, of which the temperature was so high as to raise Reaumur's thermometer to the degree of 60, equal to 86.25 cent., or 187.25 of Fahr. According to his account, one could not put one's hand in it; yet he distinctly saw fish which did not appear to be at all incommoded by the heat; and small plants, the *agnus castus,* flourishing in it.—Journal de Physique de Rosier, April 1774, p. 256. See also Rees's Cyclopædia and Pinkerton's Geography.

"The *sparus Desfontaines* of Lacepede, the *chromis* of Cuvier, was found by Desfontaines in the hot waters of Cafsa in Barbary, in which Reaumur's thermometer rose to 30 degrees. See the article 'Sparus' by Bosc, Dictionaire d'Histoire Naturelle, Deterville's edition, vol. xxxi. p. 550. My friend likewise received the same statement from the Professor's own mouth.

"The following extract is from Bruce. 'At Feriana, the ancient *Thala*, are baths of warm water without the town: in these there were a number of fish, about four inches in length, not unlike gudgeons. Upon trying the heat by the thermometer, I remember to have been much surprised that they could have existed, or even not been boiled, by continuing so long in the heat of this medium." (P.465.)

"The late lamented Baron Cuvier, whilst engaged in publishing his great work on fishes, was reminded of these observations by my friend, and in consequence wrote to M. Marcéscheau, the French vice-consul at Tunis, who not only confirmed the fact in his reply, but sent him two long-tailed fresh-water turtles, from a basin of water at Utica, of which the water is at the temperature of 36 degrees of Reaumur, or 113 of Fahr. The vice-consul also sent some fishes from the hot waters of Cafsa and Tozer, which proved to be *chromis* or *spari Desfontaines* of Lacepede. These waters were said to be as warm as 62 degrees of Reaumur.

"Breislak, in his Institutions Géologiques, has an article on this subject. Amongst other facts, to those above noticed, he adds, that Dunbar and Hunter, in their journey made in 1804, along the Washila or Ouachita, a river of Louisiana, observed above Fort Meiro, on the frontiers of the United States, springs of the temperature of 40 degrees to 50 degrees of Reaumur, or 122 to 145 Fahr., in which were not only growing *confervæ* and herbaceous plants, but also shrubs and trees. They likewise found in them *bivalve molusca.*

"Lamark, in his Histoire des Animaux sans Vertebres, states, that the *paladina muriatica* is found in Italy and in France, especially in the south, inhabiting in fresh water, and has been met with in water of the temperature of 34 degrees Reaumur, 109 Fahr."

"I have not brought forward these curious facts, with the intention of disputing the general accuracy of the limits of high temperature assigned by Dr. Edwards, as consistent with the life of fishes. As exceptions to it, they may be apparent rather than real, since it is by no means impossible, that the heat of that part of the water in which the fishes were seen, might not be exactly the same as that in which the thermometer was placed. If they cannot in this manner be explained away, they afford a very legitimate object for further inquiry. The following fact is quite compatible with the limit given by Dr. Edwards, but it is interesting, as shewing that a very considerable degree of warmth is not only supported, but very congenial to some species of fish. It is well known that, in manufacturing districts, where there is an inadequate supply of cold water for the condensation of the steam employed in the engines, recourse is had to what are called engine dams or ponds, into which the water from the steam-engine is thrown for the purpose of being cooled: in these dams, the average temperature of which is about 80 degrees, it is common to keep gold-fish, the *ciprinus aureus;* and it is a notorious fact, that they multiply in these situations much more rapidly than in ponds of lower temperature exposed to the variations of the climate. Three pair of this species were put into one of these dams, where they increased so rapidly that, at the end of three years, their progeny, which were accidentally poisoned by verdigris mixed with the refuse tallow from the engine, were taken out by wheelbarrows full. Gold-fish are by no means useless inhabitants of these dams; they consume the refuse grease which would otherwise impede the cooling of the water by accumulating on its surface. It is not improbable, that this unusual supply of aliment may cooperate with increase of temperature in promoting the fecundity of the fishes. My friend, Charles May, of Ampthill, to whom I am indebted for the fact just related, has communicated to me another fact in proof of the high temperature which vegetable life is sometimes capable of enduring. John Daulby, brother to the curator at the Botanic Garden, at Liverpool, brought from Iceland, a short time since, a species of *Chara,* which he found flowering and producing seed in one of the hot springs of that island, in which he states, that he boiled an egg in four minutes." (P. 467.)

As to the power of warm-blooded animals, at different ages, resisting the morbid influence of cold, and the effects of cold in causing death and producing disease, among much other interesting matter, we find the following, which is practically important.

"Since the publication of Dr. Edwards's observations respecting the heat of young animals, some interesting researches have been made by his brother, Dr. Milne Edwards, in conjunction with Dr. Villerme. They not only prove the inferiority of the infant's power of resisting cold, but shew, in a forcible and striking manner, the great practical importance of bearing this fact in mind. It is the custom in France to convey infants, within a few hours of their birth, to the office of the mayor of the quarter in which the nativity took place, in order that the birth may be registered, and the child become possessed of its civil rights. A careful comparison of the register of births, with the register of deaths, furnished statistical observations on so large a scale, that there can be no room to doubt the correctness of the results. It appeared that the proportion of deaths, within a very limited period after birth, compared with the total births, was much greater in winter than in summer, and that this difference of proportion was much greater in the northern and colder departments, than in the southern and warmer. The details of this investigation are recorded in a paper which the Doctors have presented to the Institute. They have since continued the inquiry, and the following extract from a letter which I have received from Dr. Milne Edwards, will shew the accordance of their results.

" 'In order to ascertain in a more positive manner than before whether the mortality of new-born children is increased by their being carried to the *maire* immediately after birth, we obtained from the minister of the interior necessary orders to have the tables of mortality of infants made in a certain number of parishes, where the inhabitants are scattered over a larger surface of ground; and in others, where they are, on the contrary, agglomerated around the *maire*. It appeared evident to us that, if our opinion was correct, the increase of mortality during winters must be much greater in the former parishes than in the latter; and such is, indeed, the result actually afforded by our tables.' " (P. 476.)

Of the influence of cold in producing disease, it will be in the memory of our readers, that a very important series of experiments and observations, made by Dr. Clendinning, appeared during the present year in several successive numbers of our Journal; and we believe the experiments there recorded are the most numerous and extensive that have been instituted, as regards the human subject: they well merit a reperusal.

In works relating to the public health, mention has been lately made of the influence of light in favoring the full, and of darkness in retarding or impairing the development of organic bodies. With regard to plants, this had long been known; but with respect to animals, and especially to man, it has only lately excited attention; and yet even already several cases are on record, in which corporeal deformities seem to be chiefly referrible to the exclusion of light. Dr. Edwards has shewn, by direct experiments, that the development of the animal body, particularly the evolution of the tadpole, from its ichthyoid to its batrachian existence, is retarded, if not prevented, by the abstraction or exclusion of light; and he concludes the chapter on this subject with the following words:

" The principles which we have deduced from experiments upon animals, lead us to the following considerations respecting man. In the climates in which nudity is not incompatible with health, the exposure of the whole surface of the body to light will be very favorable to the regular conformation of the body. This application is confirmed by an observation of Alexander de Humboldt in his voyage to the equinoctial regions. Speaking of the Chaymas, he says: 'both men and women are very muscular, their forms are fleshy and rounded. It is needless to add, that I have not seen a single individual with a natural deformity. I can say the same of many thousands of Baribs, Muyscas, and Mexican and Peruvian Indians, which we have observed during five years. Deformities and deviations are exceedingly rare in certain races of men, especially those which have the skin strongly coloured.'

"On the other hand, we must also conclude that the want of sufficient light must constitute one of the external causes which produce these deviations of form in children affected with scrofula; which conclusion is supported by the observation that this disease is most prevalent in poor children living in confined and dark streets. We may from the same principle infer, that in cases where these deformities do not appear incurable, exposure to the sun, in the open air, is one of the means tending to restore a good conformation. It is true that the light which falls upon our clothes, acts only by the heat which it occasions, but the exposed parts receive the peculiar influence of the light. Among these parts, we must certainly regard the eyes as not merely designed to enable us to perceive colour, form, and size. The exquisite sensibility to light must render them peculiarly adapted to transmit the influence of this agent throughout the system, and we know that the impression, of even a moderate light, upon these organs produces, in several acute diseases, a general exacerbation of symptoms." (P. 210.)

The last chapter (xvi.) "on the Alterations in the Air from Respiration," contains many ingenious and satisfactory experiments, which serve as the foundation of a new theory of respiration, in which that power of mixture of fluids, whether liquid or aëriform, first noticed by PORRETT, under the term of Electrofiltration, and subsequently followed out by DUTROCHET, in his treatises on Endosmose and Exosmose, and since then by STEVENS, MITCHELL, and others, plays an important part.

We regret that we can only give the "general view" with which the author concludes this chapter: the experiments would lose much of their force by desultory extracts, and they are too copious to quote at length. We shall, however, endeavour to make up for their omission, by concluding this notice with Dr. Hodgkin's note, which gives a summary account, not only of them, but also of others which have been collaterally made.

"*General View of the Alterations of the Air in Respiration.* The oxygen which disappears in the respiration of atmospheric air is wholly absorbed. It is afterwards conveyed, wholly or in part, into the current of circulation.

"It is replaced by exhaled carbonic acid, which proceeds wholly, or in part, from that which is contained in the mass of the blood.

"An animal breathing atmospheric air also absorbs azote; this is likewise conveyed wholly, or in part, into the mass of the blood.

"The absorbed azote is replaced by exhaled azote, which proceeds wholly, or in part, from the blood.

"Here are four fundamental points:

"1st. The absorption of oxygen which disappears.

"2d. The exhalation of carbonic acid which is expired.

"3d. The absorption of azote.

"4th. The exhalation of azote.

"The two first relate to the oxygen, the two others to the azote.

"According to this view, respiration is not a purely chemical process, a simple combustion in the lungs, in which the oxygen of the inspired air unites with the carbon of the blood, to form carbonic acid, to be expelled; but a function composed of several acts. On the one hand there are absorption and exhalation, attributes of all living beings; on the other the intervention of the two constituents of atmospheric air, oxygen and azote.

"This view is not a preconceived idea, but a result to which we have been necessarily led by a multitude of facts.

"It exhibits to us animated beings drawing from the composition of the atmosphere two of their constituent principles.

"It furnishes us with numerous inferences, several of which are supported by facts already received in science.

"Thus the oxygen which disappears being absorbed, and the carbonic acid exhaled, the relative proportions are necessarily variable, from the nature of the two functions which must vary in the extent of their action. The fact is beyond doubt. They may vary in three ways. 1. The carbonic acid may be expired in smaller quantity than in the oxygen which disappears; 2, in equal quantity; 3, in excess. The first is the ordinary case; the second is supported by the experiments of Allen and Pepys; the third, if it is not yet established, will probably be so hereafter. I may even say that it is so already, when we revert to the experiment of Allen and Pepys, relative to respiration in factitious air, composed of oxygen and hydrogen. The same observation applies to azote absorbed and exhaled.

"Let us return to the oxygen, and consider what becomes of it in the system. When it is absorbed and carried into the blood, there is every reason to believe, that it contributes to the formation of carbonic acid. But the experiments which I have already detailed prove that it cannot be the only source of the gas contained in the blood.

"Since we have shewn that certain species of animals can exhale in a given time as much carbonic acid in hydrogen, as in atmospheric air, there must be one or more subsidiary sources for the carbonic acid contained in the blood. It is easy to point out one. We know, from the researches of Jurine, Chevreul, Magendie, and others, that this gas exists in almost the whole extent of the alimentary canal. We cannot but admit, that it is formed in the process of digestion. It is in contact with almost the whole mucous surface of the alimentary canal, and a part must be absorbed. If any doubt of this were entertained, cases might be cited in which water impregnated with carbonic acid, and drunk in sufficient quantity, has produced symptoms of asphyxia. Doctor Desportes has communicated observations on this subject to the Royal Academy of Medicine.

"With respect to the oxygen which is to contribute to the formation of the carbonic acid contained in the mass of the blood, one of two things must happen. It enters into combination either suddenly or slowly. In the latter case there will be oxygen in excess, circulating in the mass of the blood. This pure oxygen will therefore be subject to exhalation, which will take place in the organs adapted for giving passage to it, as happens in fishes, in the air-bladders of which animals oxygen is found. I propose following up this subject, and examining different kinds of blood, in conjunction with M. Dumas." (P. 242.)

"In considering the observations of Dr. Edwards on the changes of the air in respiration, there are two points which appear to be particularly interesting and worthy of attention. The experimenters who have succeeded him had arrived at different conclusions, more especially with respect to the consumption of oxygen, and the alterations in the quantity of nitrogen. As these differences could not be attributed to errors of observation, they tended to render the subject more complex and puzzling, until Dr. Edwards, by instituting a series of experiments, continued through the different seasons of the year, at once confirmed and explained the discrepancies of his predecessors, and made a valuable discovery respecting the influence of climate and season. The other point to which I have alluded, refers to the part of the body in which the changes in the respired air are effected. It had been a subject of question, whether the carbonic acid expired was not formed immediately in the lungs, by the combination of the oxygen of the atmosphere with the carbon of the venous blood. According to another view, it was supposed that whilst a portion of the oxygen of the atmosphere was taken up by the blood, and carried with it in its circulation, and at the same time carbonic acid was thrown off from the lungs, having been previously taken up in the course of the circulation. Dr. Edwards appears to have settled this question, which seemed previously to be nearly balanced, by confirming the latter view. We have, therefore, in the function of respiration, not only a striking instance of the transudation, and imbibition of the gases through the membrane, but also of their simultaneous passage in different directions. In both of these respects, Dr. Edwards has anticipated Fodera and Dutrochet, whose observations have further elucidated them, and pointed out analogous phenomena in other parts and functions. Since the publication of Dr. Edwards's work, some further experiments on respiration have been performed by those careful and accurate operators, my friend William Allen, and his associate, W. H. Pepys; and others of equal interest, by my friend S. Broughton. Before I notice the facts, which these experimenters have either confirmed or added, it appears necessary that I should notice the discoveries and views of Dr. Stevens, which throw the most important light on the process of respiration. These views were not the offspring of speculation, which he has sought to confirm by subsequent experiments, but they forced themselves upon him, whilst he was investigating the changes of the blood, and the phenomena of fever; and it seems necessary that I should remark, to set aside any prejudice which may exist in the mind of the reader, that the Doctor's physiological observations respecting respiration stand upon their own distinct merits, and are by no means compromised by his pathological and therapeutical doctrines. In order to keep the subjects distinct, I purposely refrain from offering an opinion respecting the last-mentioned points; yet I cannot withhold the expression of my admiration of the zeal, perseverance, and self-devotion, with which the Doctor has pursued his investigations respecting them, under the most arduous, and, perhaps, perilous circumstances. One of the most striking facts which the Doctor has brought into notice, is the powerful attraction which exists between oxygen and carbonic acid. It was so fully admitted amongst chemists, that carbon in carbonic acid is united with its maximum dose of oxygen, that the idea of attraction between carbonic acid and oxygen was rejected from *à priori* reasoning by several able chemists, to whom the Doctor mentioned the subject. The fact, however, is clearly proved by the experiments of Dr. Stevens. If a receiver filled with carbonic acid, and closed by a piece of bladder firmly tied over it, be exposed to the atmospheric air, the carbonic acid, notwithstanding its superior specific gravity, rapidly escapes, and does so without the exchange of an equivalent portion of atmospheric air; the bladder is consequently forcibly depressed into the receiver. If the converse of this experiment be tried, and the receiver, containing atmospheric air, be tied over with a piece of bladder, or thin leather, and then be immersed in carbonic acid, this gas will so abundantly penetrate the membrane, and enter the receiver, as to endanger its bursting.

" Dr. Stevens had repeated opportunities of verifying these facts, during a stay which he made at Saratoga, in the United States; the springs at which place liberate a large quantity of carbonic acid. In the high rocks it often collects in considerable quantity and purity, and experiments on dogs and rabbits are often made for the entertainment of strangers, as at the Grotto del Cane, near Naples. This rock stands by itself in a low valley, through which there run two currents of water, the one fresh and superficial, the other beneath, and charged with salts and carbonic acid. A current of this water rises to some height in a cavity of the high rock, which appears to have been formed by a deposition of earthy salts from the water. It has a conical figure, the base of which is below the surface of the ground, is about nine feet in diameter. It rises above five feet from the ground, where it is truncated, and presents an aperture a foot in diameter. The cavity of the rock is conical, like its external figure, the water appears formerly to have overflowed the summit, but it now rises in general only about two feet above the ground. In the three feet above, the liberated carbonic acid collects, but it varies very much, both in quantity and purity, notwithstanding the sides of the rock are thick and impervious, and the superior specific gravity of the gas, which is constantly liberated in large quantity. The removal of the carbonic acid appears to be effected by virtue of that attraction, which Dr. Stevens has pointed out as existing between it and the oxygen of the atmosphere. When the air is somewhat agitated by wind, a taper will burn in the cavity of the rock, almost as low as the surface of the water; but when the air is calm, the taper is extinguished much nearer the top of the rock. By luting a large funnel over the aperture, so as to exclude the influence of the air, the rock became filled with carbonic acid, which the Doctor collected for his experiments at the mouth of the funnel. Dr. Stevens took advantage of the facilities afforded by this rock, to multiply and vary his experiments, the results of which were not only perfectly satisfactory to himself, but to many individuals to whom he exhibited them. This attraction, which the Doctor has pointed out, is not only to be regarded as an important agent in the function of respiration, but throws considerable light on the constitution of the atmosphere, since it accounts for carbonic acid, notwithstanding its greater specific gravity being found in equal proportions at every elevation to which we can ascend, instead of being collected at or near the surface of the earth.

" Experiments similar to those of Dr. Stevens, and attended with the same results, have been published in an American Journal, by Drs. Faust and Mitchell, who have anticipated Dr. Stevens, in committing them to the press, without making any allusion to his discovery, although there can be but little doubt but they were in a degree acquainted with it, as the Doctor himself had related the result of his previous experiments, not only to other professional individuals in the United States, but even to the very editor of the Journal in which the American papers were first published. It is stated also, that this gentleman took a part in Dr. Mitchell's experiments. Dr. Stevens formed his views, respecting the attraction of the atmosphere for carbonic acid, and committed them to paper, in 1827, at which time he resided in the West Indies. In 1828, they were mentioned, or shewn in manuscript, to several persons in this country; and in France, which the Doctor visited in 1830, more than one chemical philospher was disposed to dispute the existence of such an attraction. Dr. Edwards himself was amongst this number.

"Dr. Stevens went to the United States in 1830, in the seventh month (July). The American experiments commenced soon after, and were published before the end of the year. The reader, I trust, will allow the excellence of the principle, *suum cuique*, to be a sufficient apology for the introduction of this statement." (P. 481.)

In a former number we have recorded our opinion of Dr. Hodgkin's physiology of the Spleen, and therefore we have the less regret in now passing over that part of the Appendix; and, with regard to the other accompanying papers, as they are only collaterally connected with the chief objects of the work, they may form fit topics for a separate notice.

